# Association between the Severity of Dental Caries and the Degree of Adherence to the Mediterranean Diet in the Pediatric Population

**DOI:** 10.3390/nu14173622

**Published:** 2022-09-01

**Authors:** Laura Marqués-Martínez, Marcelino Pérez-Bermejo, Ana Rosa Lairón-Peris, Clara Guinot-Barona, Carla Borrell-García, Esther García-Miralles

**Affiliations:** 1 Dentistry Department, Faculty of Medicine and Health Sciences, Catholic University of Valencia San Vicente Mártir, 46001 Valencia, Spain; 2 SONEV Research Group, School of Medicine and Health Sciences, Catholic University of Valencia, C/Quevedo n° 2, 46001 Valencia, Spain

**Keywords:** caries, Mediterranean diet, ICCMS, KIDMED, children

## Abstract

Children who show better eating practices are less likely to suffer from severe caries than those who eat a diet rich in sugars. In the present study, we aimed to establish the relationship between the severity of dental caries and adherence to the Mediterranean diet. A cross-sectional study was carried out in which 263 children aged 2 to 14 years old were examined intraorally to analyze the presence and severity of caries. Children’s parents/caregivers completed the KIDMED questionnaire to determine their degree of adherence to the Mediterranean diet. The results showed that the prevalence of caries is greater than 80% in children with medium or low adherence to the Mediterranean diet, and remains significant at 67% in the high adherence group (*p* = 0.010). A statistically significant negative correlation of weak magnitude (r = −0.29; *p* < 0.001) was found between adherence and the number of carious teeth. Caries severity in the first molars is also influenced by adherence to the diet in a statistically significant way. In conclusion, there is an association between adherence to the Mediterranean diet and the prevalence, extension, and severity of caries in the pediatric population.

## 1. Introduction

The progression of carious lesions causes pain and ultimately tooth loss, which can affect basic physiological functions such as eating, sleeping, and speaking, and hinder general good health [1,2]. Therefore, it is necessary to treat these lesions in their early stages, strengthen community dental care programs, and implement prevention from the first year of life [3,4]. Some factors that favor the appearance of caries are difficult to change, but others such as oral hygiene and diet can be modified, especially in early childhood [5,6,7].

It has been observed that when access to sugar is limited, caries prevalence is very low [5,8]. In addition, anticariogenic properties of some food groups such as dairy, whole grains, and high-fiber fruits have been documented [9,10]. However, apart from studying the impact of individual foods, diet as a whole must also be studied [6]. This approach is innovative for coping with the disease since reducing sugar intake can be difficult [11].

Traditionally, the Mediterranean diet is known for restricted consumption of meats, sweets, and ultra-processed meats; increased consumption of plant-based foods such as fruits, vegetables, and legumes [12,13,14]; and moderate consumption of fish [15], as well as the use of extra virgin olive oil as the main source of fat [14,16,17]. However, in recent years, there has been a decline in its adherence due to globalization and changes in the agri-food industry [13,18].

Therefore, since there is evidence supporting the relationship between eating patterns and dental caries [19], and as research on the association of caries with the Mediterranean diet in particular is very limited, the aim of the present study was to analyze whether adherence to this diet can be associated with a lower prevalence or severity of dental caries in order to recommend it from an early age.

## 2. Materials and Methods

### 2.1. Study Design and Participants

This study was conducted following the STROBE standard for cross-sectional studies and approved by the ethics committee (approval code CEI20/066). The study was undertaken with the understanding and written consent of each participant’s caregivers and in accordance with the World Medical Association’s Declaration of Helsinki. Data were collected between October 2021 and May 2022.

Prior to the start of the study, protocolization was tested in 25 patients who were not included in the database, and the sample size was calculated. The test allowed the calibration of the principal investigator for the clinical diagnosis of carious lesions. In the calibration tests, an intra-examiner Kappa value of 0.95 was obtained. For the calculation of the sample size, the formula for the descriptive estimation of a single proportion with a confidence interval of 95% and a precision of 3% was used, concluding that a sample size of 203 patients will be needed.

Lastly, a total of 263 patients between 2 and 14 years of age who attended a dental clinic in Valencia to receive pediatric dental treatment were selected. During the first visit, it was evaluated whether the patient met the inclusion criteria to participate in the research. These criteria were that patients were attending a dentist for the first time ever, patients had reached the age of 2 but not the age of 15, and parents/caregivers had signed the informed consent and filled out the study questionnaire correctly and completely. The exclusion criteria were patients presenting physical, mental, or sensory disabilities that prevented the performance of the clinical examination, along with being carriers of orthodontic appliances or space maintainers, presenting any enamel defect both of genetic and environmental origin, and the presence of dental fractures.

When a patient met the criteria, a member of the research team explained the purpose of the study to parents/caregivers and asked if they wanted the patient to participate anonymously, voluntarily, and without any financial compensation. In the case of an affirmative answer, informed consent was given and the instructions for the KIDMED [20] questionnaire were explained in order to determine the degree of adherence of the child or adolescent to the Mediterranean diet.

While the parent/caregiver completed the KIDMED questionnaire in the waiting room, the main investigator examined the present teeth in each patient’s mouth and recorded the obtained value of caries activity and severity according to the International Caries Classification and Management System (ICCMS) [21].

### 2.2. Variables

The following data were collected from each patient who participated in the study: age (months); sex (male or female); degree of adherence to the Mediterranean diet using the KIDMED questionnaire completed by the father, mother, or legal guardian of the minor; and ICCMS value for each temporary and/or permanent tooth present in the patient’s mouth.

Adherence to the Mediterranean diet was derived from the KIDMED questionnaire developed in 2004 by Serra-Majem et al. [20], which is a scoring instrument used in children and young people to determine their adherence to the Mediterranean diet through a questionnaire with 16 yes or no questions. The questions are valued with a positive point (+1) if they are close to the Mediterranean diet model and with a negative point (−1) if they are far from it. Numerical assessment ranges are between 0 (minimum adherence) and 12 (maximum adherence), establishing three ranges: optimal Mediterranean diet or high adherence to the Mediterranean pattern (score ≥ 8), need to improve the eating pattern to adjust it to the Mediterranean model or average adherence (score 4–7), and very low-quality diet or low adherence to the Mediterranean pattern (score ≤ 3).

In order to determine the prevalence, severity, and extent of carious lesions, a clinical examination was performed on each patient using a No. 5 flat mirror (Hu-Friedy, Rotterdam/The Netherlands) and a WHO 11.55 B periodontal probe (Hu-Friedy, Rotterdam/The Netherlands). The ICCMS value for each of the teeth present in the mouth was recorded using the ICCMS Caries Classification established by the ICCMS™ Guide published in 2014 [21]. According to this diagnosis method, teeth must first be explored wet and subsequently dried with an air syringe for five seconds to be re-examined and thus establish a definitive diagnosis. Each tooth was assigned a value from 0 to 6, where 0 = healthy tooth surface; 1 = initial visual change in enamel after drying; 2 = initial visual change in enamel with wet tooth; 3 = fracture located in the enamel without exposed dentin; 4 = underlying dark shade of dentin with or without enamel loss, no exposed dentin; 5 = cavity with exposed dentin occupying less than 50% of the surface; and 6 = cavity with exposed dentin occupying more than 50% of the surface.

### 2.3. Statistical Analysis

Statistical analysis was performed using the IBM SPSS Statistics 15.0 statistical package to study the association between caries prevalence/severity variables and adherence to the Mediterranean diet.

The chi-squared test was used to evaluate the homogeneity of caries prevalence in the three levels of adherence to the Mediterranean diet. The Kruskal–Wallis test was used to compare the distribution of the ICCMS grade in the three levels of adherence to the Mediterranean diet. Pearson’s correlation was used to assess the degree of linear association between the KIDMED score and the number of teeth with caries. Spearman’s nonlinear correlation was used for the KIDMED score and ICCMS for each tooth.

The level of significance used in the analysis was 5% (α = 0.05). Any *p*-value less than 0.05 is indicative of a statistically significant relationship. In contrast, a *p*-value greater than or equal to 0.05 indicates no relationship.

To estimate the population prevalence from the sample of 263 individuals, there is a maximum error of 6% for *p* = *q* = 5% and a confidence level of 95%. For a chi-squared test in the current sample, with a confidence level of 95%, the power reached is 81.4% to detect two different caries prevalence rates, 70% and 85%, in two groups (such as medium and high adherence) as significantly different.

## 3. Results

### 3.1. Sex and Age of the Sample

The sample consisted of 263 children, of whom 130 were male (49.4%) and 133 were female (50.6%), with an overall mean age of 7.3 ± 2.2 years and a range between 2 and 14 years of age.

### 3.2. Prevalence and Extension of Carious Lesions

Sixty-five individuals (24.7%) did not present caries in any of their teeth, making the caries prevalence in the sample 75.3% (95% CI: 70.1–80.5%). Furthermore, on average, one individual in the sample had 4.0 ± 3.8 teeth affected by caries.

**Figure 1:**
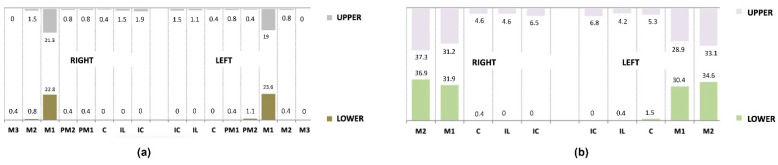
Caries prevalence (%): (**a**) permanent dentition; (**b**) primary dentition.

Figure 1 shows the caries prevalence in the total sample for each of the teeth of the primary and permanent dentition.

### 3.3. Adherence to Mediterranean Diet

**Figure 2:**
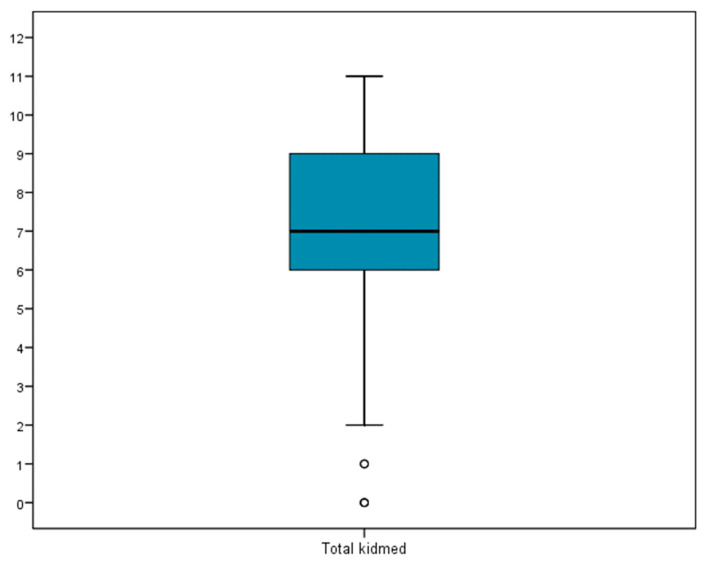
Distribution of KIDMED questionnaire values.

On average, the KIDMED score was 7.2 ± 2.2. The median of 7 means that half of the patients presented a score equal to or greater than 7. The IQR 6–9 interquartile range means that half of the sample scored between 6 and 9 (Figure 2). In total, 48.3% of the children presented high adherence to the Mediterranean diet, 46.4% presented medium adherence, and only 5.3% presented low adherence.

### 3.4. Association between Caries and Adherence to the Mediterranean Diet

#### 3.4.1. Prevalence of Dental Caries and Adherence to Mediterranean Diet

**Figure 3:**
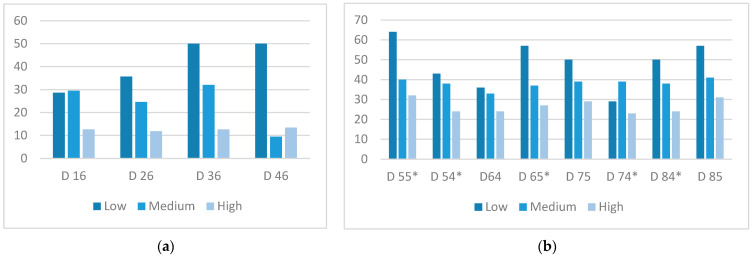
Caries prevalence of the most relevant teeth according to the level of adherence to the Mediterranean diet: (**a**) permanent dentition; (**b**) temporary dentition. * Statistically significant differences.

Table 1 shows the results for the prevalence of caries in any permanent and temporary tooth present in the mouth according to adherence to the Mediterranean diet. In all permanent teeth, except for the first molars, the prevalence of caries was very low or could not be assessed due to lack of tooth eruption; thus, many of the tests could not be applied or failed to provide evidence. However, statistically significant differences were detected in the first four permanent molars. The caries rate progressively decreased as adherence to the Mediterranean diet increased. For instance, for tooth 4.6, caries was detected in 50% of children with low adherence, in 29.5% with medium adherence, and only in 13.4% with high adherence (Figure 3). In the primary dentition, the differences reached statistical significance only in some teeth. Figure 3 shows the results for the teeth with the highest caries prevalence.

#### 3.4.2. Global Caries Prevalence and Adherence to the Mediterranean Diet

Table 2 shows the global prevalence of caries according to adherence to the Mediterranean diet. The prevalence of caries surpassed 80% in children with medium or low adherence to the Mediterranean diet, while it remained 67% in the high adherence group. This contrast between both rates is statistically significant (*p* = 0.010).

#### 3.4.3. Caries Extension (Number of Affected Teeth) and Adherence to Mediterranean Diet

Table 3 shows clear differences in terms of the mean number of affected teeth based on adherence to the Mediterranean diet.

If this number is directly compared with the actual KIDMED score, a clearly negative slope is observed: the greater the adherence, the less decayed are teeth. The correlation is weak in magnitude (r = −0.29) but statistically significant (*p* < 0.001).

#### 3.4.4. Caries severity (ICCMS) and Adherence to the Mediterranean Diet

Table 4 shows the results for the association between caries severity according to the tooth and the KIDMED score. For the permanent dentition, the significant correlations are almost restricted to the first molars, as occurred in the previous prevalence study. For the primary dentition, results are also similar to the prevalence study.

## 4. Discussion

Early childhood caries is a serious form of dental caries that affects children in the early years of life. Its etiology depends on many factors. One risk factor for the development of caries in children is diet [22]. Therefore, in the present study, we investigated the relationship between adherence to the Mediterranean diet and dental caries in children.

Dental caries is highly prevalent. In this study, it affected 198 individuals in the sample, thus having a prevalence of 75.3%. However, it is difficult to compare this result with those published in other articles because although the term early childhood caries is widely used, various authors use different diagnostic criteria and samples [23].

Caries is believed to affect 80–90% of the world’s population [24,25], and according to the World Health Organization (WHO), between 60% and 90% of the world’s children have dental caries [26], a range within which our sample falls. However, in the most recent 2018 epidemiological survey of caries in children and young people in the Valencian Community [4], it was found that the prevalence of caries in children in the Valencian Community was 37.4% and the rate of decayed and filled teeth was 1.23 at 6 years of age for primary dentition. For permanent dentition at 12 years of age, the caries prevalence was 30.1% with an index of decayed, filled, and missing teeth of 0.66. In permanent dentition at 15 years of age, the prevalence was 44.6%, and the index of decayed, filled, and missing teeth was 1.21. These results differ from ours because to calculate the prevalence in the 2018 survey, caries was considered from ICDAS code 4 (moderate stage of caries, localized caries rupture and underlying dentin shadow). We, on the other hand, consider its presence from ICDAS code 1 since the ICCMS™ Guide considers it the initial stage of caries [27,28].

The KIDMED questionnaire is a reliable instrument to assess the degree of adherence to the Mediterranean diet in schoolchildren [29] and is one of the most used scoring systems for this purpose [30]. Our investigation concluded that 48.3% of the sample presented high adherence, 46.4% presented medium adherence, and only 5.3% showed low adherence. In other studies carried out in Spain in which the KIDMED questionnaire was used [20,31,32], it was most common for patients to present a high or moderate adherence to the Mediterranean diet.

However, despite the fact that the KIDMED questionnaire has been used in many studies both in Spain [31,32,33,34,35,36] and in other countries [6,29,37,38,39], the purpose for which it has been used in each of them is different. The association between the Mediterranean diet and different variables such as obesity [34,38], allergic/respiratory morbidity [37], menstrual pain [35], or recommendations for physical activity and screen time [32], among others, has been studied. The association between the Mediterranean diet and caries, as in our work, has been scarcely investigated [36], with the articles by Inan-Eroglu et al. [6] and Marqués-Martínez et al. [36] being the only publications on the subject.

Inan-Eroglu et al. [6] examined the association between the Mediterranean diet and dental caries in 395 children between 36 and 71 months of age from Ankara using the Healthy Eating Index-2010 (HEI-2010), the KIDMED questionnaire, the WHO 2013 criteria, and the ICCMS criteria. It was concluded that although children with low KIDMED scores had slightly higher values of decayed, filled, and missing teeth compared to children with good or average scores, the differences were not statistically significant. Contrary to the KIDMED findings, the mean value of dental caries was significantly higher among children with a poor HEI-2010 score compared to children with a medium HEI-2010 score.

Marques et al. [36] studied the relationship between caries and adherence to the Mediterranean diet in a sample of 268 Valencian children between 3 and 9 years old using the KIDMED questionnaire and the cod and cos indices. In this case, a statistically significant relationship was observed between these indices and low adherence to the Mediterranean diet.

Both studies coincide with ours in that there is a correlation between the Mediterranean adherence and caries. The greater the adherence to the Mediterranean diet, the lower the amount of caries. The less adherence to the Mediterranean diet, the greater the amount of caries. The hypothesis raised at the beginning of the investigation is not only accepted as a result of our findings, but it is also supported by other authors.

This can be explained by the fact that the Mediterranean diet is characterized by high consumption of plant-based foods (vegetables, fruits, nuts, legumes, and unprocessed cereals), moderate to high consumption of fish, low consumption of meat (especially red and processed meats), low consumption of dairy (with the exception of yogurt and maturated cheeses), and occasional consumption of ultra-processed or high sugar content products. In addition, olive oil is the main source of fat for cooking and the key to the Mediterranean diet [40].

Polyols or polyalcohols, which are found naturally in plant-based food such as fruits and vegetables [41], are considered anticariogenic agents [42,43] since they favor an increase in salivary pH and thus remineralize carious lesions in their initial stages [44]. Furthermore, some polyols such as xylitol inhibit bacterial growth [44,45]. In addition, phenolic compounds found in fruits and vegetables reduce the growth of bacteria related to caries and the formation of biofilm [46] and have an inhibitory effect on the enzymatic activity of glycosyltransferases, the proteins that can catalyze the reaction for the metabolism of sucrose [47].

Cheese and yogurt have anticariogenic properties, as well. Several mechanisms have been proposed to explain this, although the most popular ones focus on the fact that milk proteins have a buffer effect on acid formation and promote salivary clearance [9].

Furthermore, ultra-processed products, carbonated drinks, or industrial pastries which are high in added sugars are almost excluded from the Mediterranean diet. Caries is a sugar-dependent disease; therefore, the exclusion of such products prevents the onset of the disease [48,49]. In fact, the literature suggests that caries is reduced whenever free sugar intake is reduced to levels below 10% of dietary energy, and that further reductions may produce additional benefits [49].

However, besides following the Mediterranean diet and promoting healthy eating habits, more strategies must be implemented to stop the appearance of dental caries. Necessary elements to improve children’s oral health include proper tooth brushing with adequate fluoride content [50], the use of dental floss, the use of chemical agents such as chlorhexidine, if indicated [51], and the administration of probiotics that compete against cariogenic bacteria by inhibiting the fermentation of sugar [52], among others.

Ultimately, although it is necessary to promote good diet patterns such as the Mediterranean diet for the prevention of early childhood caries [6,20], the role of diet in caries should not only focus on the foods that can cause it, but also on those that protect against its appearance. There is a need to incorporate a more holistic view of diet and nutrition as they relate to caries since reducing sugar intake can be a difficult goal to achieve; however, emphasizing foods that can protect against caries results in a novel approach to deal with the disease [11].

## 5. Conclusions

This work shows a clear association between adherence to the Mediterranean diet and the prevalence, extension, and severity of caries in the pediatric population. Further studies which correlate the Mediterranean diet and dental caries in childhood should be carried out in order to develop dietary strategies aimed at prevention.

## Figures and Tables

**Table 1:** Caries prevalence for each tooth and adherence to the Mediterranean diet (medium–low vs. high).

Permanent Tooth	*p*-Value	Primary Tooth	*p*-Value
1.7	0.123	5.5	0.037 *
1.6	0.001 **	5.4	0.035 *
1.5	0.499	5.3	0.031 *
1.4	0.499	5.2	0.018 *
1.3	1.000	5.1	0.264
1.2	0.623	6.1	0.183
1.1	0.372	6.2	0.029 *
2.1	0.623	6.3	0.137
2.2	1.000	6.4	0.292
2.3	1.000	6.5	0.034 *
2.4	1.000	7.5	0.137
2.5	1.000	7.4	0.027 *
2.6	0.004 **	7.3	0.891
2.7	0.499	7.2	0.560
3.7	1.000	7.1	-
3.6	<0.001 ***	8.1	-
3.5	0.248	8.2	-
3.4	1.000	8.3	0.548
3.3	-	8.4	0.026 *
3.2	-	8.5	0.066
3.1	-		
4.1	-		
4.2	-		
4.3	-		
4.4	1.000		
4.5	1.000		
4.6	<0.001 ***		
4.7	1.000		

* *p* < 0.05; ** *p* < 0.01; *** *p* < 0.001.

**Table 2:** Caries prevalence according to adherence to the Mediterranean diet.

	Adherence to Mediterranean Diet							
	Total		Low		Medium		High	
	*n*	%	*n*	%	*n*	%	*n*	%
Total	263	100%	14	100	122	100%	127	100%
No	65	24.7%	2	14.3%	21	17.2%	42	33.1%
Yes	198	75.3%	12	85.7%	101	82.8%	85	66.9%

**Table 3:** Number of teeth presenting caries according to adherence to the Mediterranean diet.

	Adherence to Mediterranean Diet			
	Total	Low	Medium	High
*n*	263	14	122	127
Mean	4.0	6.1	5.0	2.9
Typical deviation	3.8	4.4	4.1	3.1
Minimum	0	0	0	0
Maximum	17.0	12.0	17.0	13.0
25th Percentile	1.0	3.0	1.0	0
Median	3.0	5.5	4.0	2.0
75th Percentile	6.0	11.0	8.0	4.0

**Table 4:** Results for the association between caries severity by tooth and the KIDMED score.

Permanent Tooth	r	*p*-Value	Primary Tooth	r	*p*-Value
1.7	−0.14	0.027 *	5.5	−0.17	0.005 **
1.6	−0.19	0.002 **	5.4	−0.16	0.009 **
1.5	−0.02	0.735	5.3	−0.13	0.031 *
1.4	−0.02	0.735	5.2	−0.13	0.038 *
1.3	−0.02	0.811	5.1	−0.08	0.187
1.2	−0.02	0.745	6.1	−0.10	0.097
1.1	−0.03	0.692	6.2	−0.12	0.062
2.1	−0.02	0.745	6.3	−0.08	0.178
2.2	−0.02	0.810	6.4	−0.12	0.049 *
2.3	−0.02	0.810	6.5	−0.20	0.001 **
2.4	0.01	0.913	7.5	−0.18	0.004 **
2.5	−0.02	0.811	7.4	−0.20	0.001 **
2.6	−0.20	0.001 **	7.3	−0.01	0.815
2.7	−0.06	0.304	7.2	−0.01	0.811
3.7	−0.08	0.227	7.1	−	−
3.6	−0.24	<0.001 ***	8.1	−	−
3.5	−0.03	0.678	8.2	−	−
3.4	−0.02	0.811	8.3	0.02	0.695
3.3	−	−	8.4	−0.20	0.001 **
3.2	−	−	8.5	−0.16	0.010 *
3.1	−	−			
4.1	−	−			
4.2	−	−			
4.3	−	−			
4.4	−0.02	0.811			
4.5	−0.02	0.811			
4.6	−0.24	<0.001 ***			
4.7	−0.04	0.560			

* *p* < 0.05; ** *p* < 0.01; *** *p* < 0.001.
